# The Powdery Mildew Effector CSEP0027 Interacts With Barley Catalase to Regulate Host Immunity

**DOI:** 10.3389/fpls.2021.733237

**Published:** 2021-09-09

**Authors:** Hongbo Yuan, Cong Jin, Hongcui Pei, Lifang Zhao, Xue Li, Jiali Li, Wanting Huang, Renchun Fan, Wende Liu, Qian-Hua Shen

**Affiliations:** ^1^State Key Laboratory of Plant Cell and Chromosome Engineering, Institute of Genetics and Developmental Biology, Innovation Academy for Seed Design, Chinese Academy of Sciences (CAS), Beijing, China; ^2^CAS Center for Excellence in Biotic Interactions, University of Chinese Academy of Sciences, Beijing, China; ^3^School of Life Sciences, Yunnan University, Kunming, China; ^4^State Key Laboratory for Biology of Plant Diseases and Insect Pests, Institute of Plant Protection, Chinese Academy of Agricultural Sciences (CAS), Beijing, China

**Keywords:** powdery mildew, *Blumeria graminis*, effector, CSEP, virulence, barley catalase

## Abstract

Powdery mildew is one of the most important fungal pathogen diseases. The genome of barley mildew fungus, *Blumeria graminis* f. sp. *hordei* (*Bgh*), encodes a large number of candidate secreted effector proteins (CSEPs). So far, the function and mechanism of most CSEPs remain largely unknown. Here, we identify a *Bgh* effector CSEP0027, a member of family 41, triggering cell death in *Nicotiana benthamiana*. CSEP0027 contains a functional signal peptide (SP), verified by yeast secretion assay. We show that CSEP0027 promotes *Bgh* virulence in barley infection using transient gene expression and host-induced gene silencing (HIGS). Barley catalase *Hv*CAT1 is identified as a CSEP0027 interactor by yeast two-hybrid (Y2H) screening, and the interaction is verified in yeast, in *vitro* and *in vivo*. The coexpression of CSEP0027 and *Hv*CAT1 in barley cells results in altered localization of *Hv*CAT1 from the peroxisome to the nucleus. Barley stripe mosaic virus (BSMV)-silencing and transiently-induced gene silencing (TIGS) assays reveal that *Hv*CAT1 is required for barley immunity against *Bgh*. We propose that CSEP0027 interacts with barley *Hv*CAT1 to regulate the host immunity and likely reactive oxygen species (ROS) homeostasis to promote fungal virulence during barley infection.

## Introduction

Powdery mildews are widespread fungal diseases that affect more than 10,000 plant species, such as important cereal crops, economic, and ornamental plants (Glawe, 2008; Dean et al., 2012; Takamatsu, 2013). As obligate biotrophic pathogens, powdery mildew fungi totally depend on the living plant cells for survival and reproduction. Mildew conidiospores attach to the epidermal tissue of the host, germinate and produce fungal infection structures, such as the appressorium and penetration peg to penetrate the plant cell wall, subsequently, the haustoria are developed within the lumen of the host cells but separated from the host cell cytoplasm by extrahaustorial membrane (EHM) and extrahaustoral matrix (EHMX) (Panstruga, 2003; Both et al., 2005). The haustorium is believed to be a site for nutrient uptake and signaling exchange (Panstruga and Dodds, 2009; Stergiopoulos and de Wit, 2009), and effector proteins are believed to deliver into the plant cells through haustorium to promote fungal virulence.

*Blumeria graminis*, the powdery mildew fungus causing disease on the cereal crop species and grasses (Poaceae), has been classified into at least eight *formae speciales* (f.sp.), each adapted to a host genus (Troch et al., 2014). *B. graminis* f.sp. *hordei* (*Bgh*) and *B. graminis* f.sp. *tritici* (*Bgt*) colonize barley and wheat, respectively. The *Bgh* and *Bgt* genomes code for ∼700 and 800 candidate secreted effector proteins (CSEPs), respectively (Godfrey et al., 2010; Spanu et al., 2010; Pedersen et al., 2012; Wicker et al., 2013; Frantzeskakis et al., 2018; Müller et al., 2019). Many *Bgh* CSEPs are overlapped with the so called *Blumeria* effector candidate (BEC) proteins identified from the proteomic analyses (Bindschedler et al., 2009, 2016; Godfrey et al., 2009). A majority of these *Bgh* CSEPs have a predicted amino-terminal signal peptide (SP) and a putative Y/F/WxC motif (Bindschedler et al., 2009; Godfrey et al., 2010; Spanu et al., 2010; Pedersen et al., 2012). A large proportion of *Bgh* CSEPs (c. 25%) are structurally predicted similar to RNase and/or RNA-binding activity, and these CSEPs are termed as RNase Like Proteins expressed in Haustoria (RALPHs) (Pedersen et al., 2012; Spanu, 2017). Interestingly, most of the so far identified *Bgh* AVR_*A*_ effectors, each recognized by a cognate barley MLA receptor, are also RALPHs with fungal RNase folds but lacking the residues required for RNase activity (Lu et al., 2016; Saur et al., 2019; Bauer et al., 2021). So far, several *Bgh* CSEPs/BECs have been functionally characterized with respect to fungal virulence through transient gene expression and host-induced gene silencing (HIGS) approaches (Bindschedler et al., 2009; Godfrey et al., 2009; Nowara et al., 2010; Spanu et al., 2010; Pedersen et al., 2012; Pliego et al., 2013; Ahmed et al., 2015, 2016; Menardo et al., 2017; Frantzeskakis et al., 2018; Pennington et al., 2019; Li et al., 2021). The host targets have been identified for some CSEPs that are involved in plant immunity and stress responses (Zhang et al., 2012; Schmidt et al., 2014; Ahmed et al., 2015; Pennington et al., 2016, 2019; Saur et al., 2019). Recently, few *Bgh* CSEPs have been showed or proposed to play a role in regulating the host cell death (Pennington et al., 2019; Li et al., 2021). A CSEP0064/BEC1054, one of the *Bgh* RALPHs, binds to RNA and may act as a pseudoenzyme to inhibit the action of the host ribosome-inactivating proteins (RIPs) that would otherwise induce cell death (Pennington et al., 2019). The CSEP0139 and CSEP0182 are capable of suppressing programmed cell death (PCD) induced by various cell death inducers in plant cells (Li et al., 2021). Despite these intensive studies, the function and mode of action of many CSEPs remain largely unclear.

Reactive oxygen species (ROS), produced from aerobic metabolism in plants, have been appreciated as major signaling molecules in plant development and in response to the biotic and abiotic stresses (Apel and Hirt, 2004; Nanda et al., 2010; Waszczak et al., 2018). In plant–pathogen interactions, ROS can directly kill the invading pathogens and trigger cell death to stop pathogen invasion, or can serve as signaling molecules to regulate the plant defense responses (Mittler et al., 2011; Mittler, 2017). Hydrogen peroxide (H_2_O_2_) and superoxide anion (O_2_^–^) are the two major ROS molecules accumulating in the plants in response to the pathogen infections. The plants rely on an intricate network to control the levels of ROS at different subcellular compartments (Hückelhoven and Kogel, 2003; Nanda et al., 2010; Petrov and Van Breusegem, 2012). Catalases are part of “the ROS network,” playing a central role in maintaining the cellular H_2_O_2_ balance and in signaling crosstalk (Du et al., 2008; Chaouch et al., 2010; Nanda et al., 2010; Sharma and Ahmad, 2014; Li et al., 2015; Zhang et al., 2015; Murota et al., 2017; Yuan et al., 2017; Chen and Jarosz, 2020; Chen et al., 2020).

In barley/wheat response to *B. graminis* infection, ROS are involved in immune responses at early and late stages of the pathogen infections (Hückelhoven and Kogel, 2003). In barley under attack by *Bgh* or *Bgt* spores, H_2_O_2_ is detected to locally accumulate in papillae (cell wall appositions) or in the whole cell, which is generally associated with host cell inaccessibility (Thordal-Christensen et al., 1997; Hückelhoven et al., 1999, 2001, 2003). The ROS are also detected in *Bgt*-attacked wheat epidermal cells and are involved in both pattern-triggered immunity (PTI) and effector-triggered immunity (ETI; Altpeter et al., 2005; Schweizer, 2008; Chang et al., 2019). On the other hand, superoxide radical anion (O_2_^–^) is believed to act in restricting cell death. In barley epidermal cells under attack by *Bgh* spores, O_2_^–^ accumulation is strictly associated with a successful penetration and O_2_^–^ also accumulates in the living cells neighboring the HR cells (Hückelhoven and Kogel, 1998; Hückelhoven et al., 2000). These studies suggest that ROS play a complex role in the plant–biotrophic fungal interactions, not only in early cell wall-associated defense and in late defense signaling but also in the cell-death suppression.

In this study, we screen ∼100 *Bgh* CSEPs through agroinfiltration in *Nicotiana benthamiana* and identify CSEP0027 triggering cell death. We show that CSEP0027 promotes fungal virulence in barley infection. We further identify CSEP0027 interactors by yeast two-hybrid (Y2H) screening and barley *Hv*CAT1 is shown to interact with CSEP0027 in yeast, *in vitro* and *in vivo*. Coexpression of CSEP0027 and *Hv*CAT1 in barley cells induces the nuclear accumulation of *Hv*CAT1 that is normally localized to the peroxisome. The functional analyses indicate that *Hv*CAT1 is involved in barley immunity against *Bgh*. We propose CSEP0027 target barley *Hv*CAT1 to regulate host immunity and promote fungal virulence in barley infection.

## Results

### CSEP0027 Specifically Induces Cell Death in *N. benthamiana*

**FIGURE 1:**
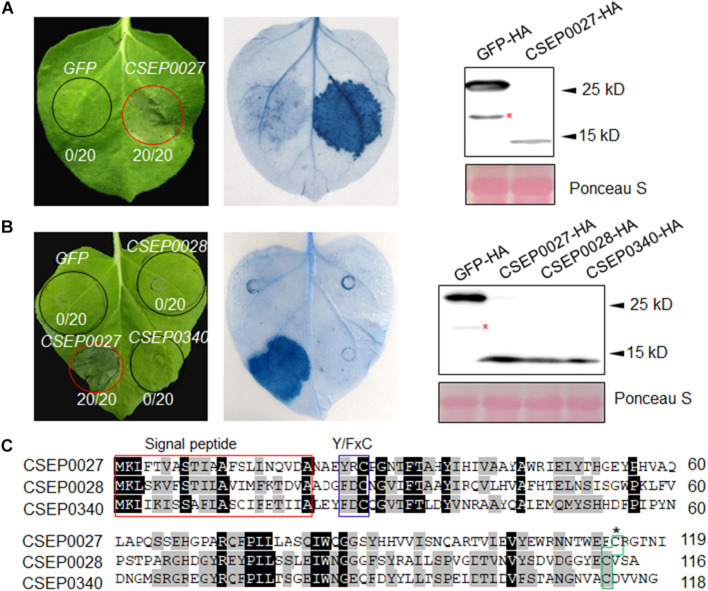
*CSEP0027* triggers cell death in *Nicotiana benthamiana*. **(A)** Expression of *CSEP0027* triggers cell death in *N. benthamiana*. *Agrobacterium tumefaciens* was used to transiently express *CSEP0027* or *GFP* in *N. benthamiana* leaves. The picture was taken at 5 dpi, and cell death was visualized by trypan blue staining. The numbers in each circled area indicate numbers of cell death in total number of leaf areas infiltrated with the construct. Total protein extract was obtained from *N. benthamiana* leaves at 60 hpi and protein expressions were confirmed by immunoblotting using anti-HA antibody. Ponceau staining was used to show equal loading. **(B)** *CSEP0028* and *CSEP0340* do not trigger cell death in *N. benthamiana*. The experimental procedures are the same as in **(A)**. The stars in the Western blots in panels **(A,B)** indicate non-specific signals. **(C)** Sequence alignment of CSEP0027, CSEP0028, and CSEP0340, performed using the DNAMAN software. The signal peptides are highlighted in red box, Y/FxC motif in blue box, and C-terminal conserved cysteine in green box.

The *Bgh* genome encodes several hundreds of potential effectors, and ∼491 effector-like proteins were initially identified to be CSEPs (Spanu et al., 2010; Pedersen et al., 2012). We selected a hundred of these *CSEP* genes for further characterization based on their expression levels and abundance in haustoria (Godfrey et al., 2010; Pedersen et al., 2012). The cDNA sequences of 101 *CSEPs* from 34 families were amplified with specific primers using RNA samples derived from barley leaf materials infected with the compatible isolate *Bgh*A6 (Supplementary Table 1). All *CSEP* cDNA sequences excluding the predicted signal peptide (ΔSP) were subcloned into vector pGR107 for *Agrobacterium tumefaciens*-mediated transient expression in *N. benthamiana* (Wang et al., 2011). We identified several CSEPs suppressing cell death in plants (Li et al., 2021), but much fewer CSEPs inducing cell death. As shown in Figure 1A, CSEP0027 is one of the CSEPs inducing clear water-soaked-like cell death phenotype in *N. benthamiana*, as compared to GFP alone, which serves as a negative control. The AVR_*a13*_ effector and its cognate receptor MLA13 were also coexpressed and triggered cell death in *N. benthamiana* (Lu et al., 2016), which severed as a positive and technique control here (Supplementary Figure 1). Trypan blue staining confirmed the localized cell death and immunoblotting verified the expression of the HA-tagged fusion proteins (Figure 1A), and DAB (3, 3′-diaminobenzidine) staining also revealed H_2_O_2_ accumulation in the infiltrated area (Supplementary Figure 2).

The CSEP0027, CSEP0028, and CSEP0340 are the three members from the same *Bgh* CSEP family 41 (Pedersen et al., 2012), in addition, BgtE-10117 and BgtE-20000 are the two potential *Bgt* homologs being identified as highly related sequences to CSEP0027 (Supplementary Figure 3A) (Praz et al., 2017). All these five CSEPs harbor a predicted SP, a Y/FxC motif, and a conserved C-terminal cysteine, with some conserved residues in the middle (Figure 1C and Supplementary Figure 3A). We tested if any of the other four CSEPs trigger cell death, unexpectedly none of them induced cell death in *N. benthamiana* (Figure 1B and Supplementary Figure 3B). CSEP0027, thus represents a unique *Bgh* effector protein to induce cell death in *N. benthamiana*.

### CSEP0027 Is a Secreted Protein

**FIGURE 2:**
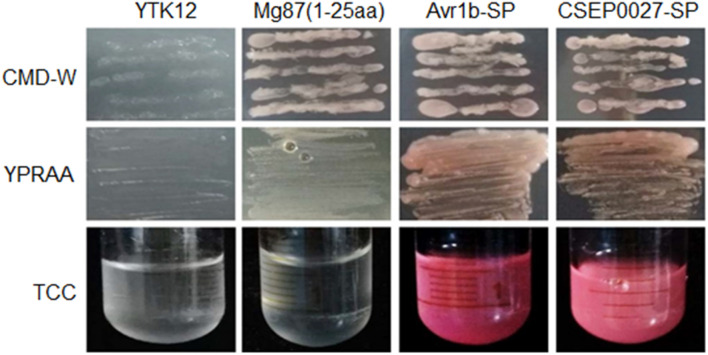
CSEP0027 is a secreted protein. Yeast invertase secretion assay was used to confirm the function of the predicted SP of CSEP0027. A construct expressing a fusion of SP sequence of CSEP0027 and a yeast invertase was transformed into the yeast strain YTK12 and tested in the assay, with the N-terminal sequence of *Magnaporthe oryzae* Mg87 protein and SP sequence of *Phytophthora sojae* PsAvr1b used as negative and positive controls, respectively. CMD-W plates were used to select yeast strain YTK12 carrying the pSUC2 vector. YPRAA media were used to indicate invertase secretion. An enzymatic activity test based on the reduction of 2, 3, 5-triphenyltetrazolium chloride (TTC) to red-colored formazan was also used to confirm invertase secretion.

To validate the secretory function of the CSEP0027 signal peptide, we used a yeast secretion assay based on invertase secretion and yeast growth on sucrose or raffinose media (Lee et al., 2006; Oh et al., 2009). The predicted SPs were fused in frame to the mature sequence of yeast invertase in the vector pSUC2 and expressed in the invertase mutant yeast strain YTK12 that otherwise cannot grow on YPRAA medium (Gu et al., 2011). CSEP0027-SP derived construct enabled transformed yeast cells to grow on YPRAA plate (with raffinose instead of sucrose as the carbon source), and so did the PsAvr1b-SP from the oomycete Avr1b effector as a positive control (Figure 2, middle panel). The first 25 amino acids of Mg87, a *Magnaporthe grisea* cytoplasmic protein as a negative control, did not enable yeast to grow (Figure 2). In addition, the secretion of the invertase was confirmed by the conversion of 2, 3, 5-triphenyltetrazolium chloride (TTC) to the insoluble red-colored triphenylformazan (Figure 2, bottom panel). These results suggest that CSEP0027 is a secreted protein carrying a functional SP.

### CSEP0027 Contributes to *Bgh* Virulence

**FIGURE 3:**
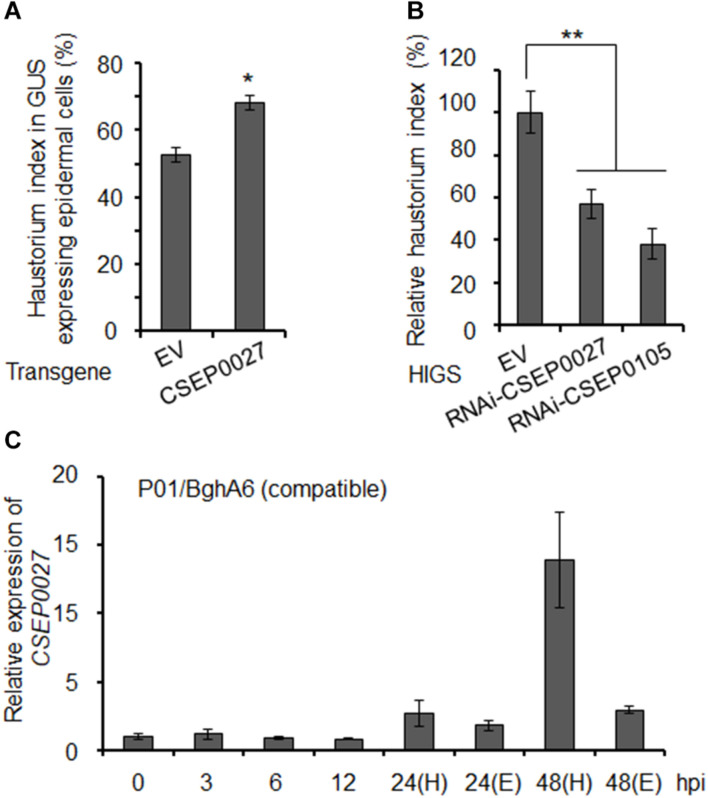
CSEP0027 contributes to *Bgh* virulence. **(A)** Overexpression of *CSEP0027* promotes *Bgh* haustorial formation rate. One-week-old barley leaves (P01) were bombarded with EV or CSEP0027 construct plus GUS reporter construct, and inoculated with compatible isolate *BghA6*. *Bgh* haustorium was microscopically scored, and haustorium index (HI%) was calculated as the number of cells containing haustorium in glucuronidase (GUS) expression cells divided by the total number of GUS expression cells with germinated *Bgh* spores and an attached appressorium. Data show the average values and SD are from three representative experiments. **(B)** Silencing of *CSEP0027* by host induced gene silencing (HIGS) reduces *Bgh* haustorial formation rate. One-week-old barley leaves (P01) were bombarded with indicated construct plus GUS reporter construct. The bombarded leaves were inoculated with the virulent isolate *BghA6* at 48 h after bombardment. *Bgh* haustorium index was microscopically scored at 48 hpi, and the relative *Bgh* haustorium index in silencing experiment was standardized to EV (pIKP007) control, which was arbitrarily set to 100%. Data show the average values and SD from three representative replicates. RNAi-CSEP0105 is used as a positive control. *p < 0.05 and **p < 0.01; show significant difference by Student’s *t* test. **(C)** Expression pattern of *CSEP0027* at early stages of *Bgh* infection. The barley P01 was inoculated with the virulent isolate *BghA6*. Total RNA was isolated from *Bgh*-infected barley leaves at 0, 3, 6, 12, 24, and 48 hpi for quantitative real-time PCR (qRT-PCR) analysis. H denotes leaf samples containing haustorium, and E denotes epiphytic *Bgh* tissues removed from the leaf surface. Relative expression was determined by comparing with time point 0 hpi, arbitrarily set to 1. *Bgh* glyceraldehyde 3-phosphate dehydrogenase was used as the reference gene. Error bars indicate SD of three replicates. The experiments were repeated two times with similar results.

To investigate the function of CSEP0027 in fungal virulence, we first overexpressed CSEP0027 in barley epidermal cells through single-cell transient gene expression followed by *Bgh*A6 infection in a compatible interaction (Bai et al., 2012). The expression of mature CSEP0027 (CSEP0027^Δ*SP*^) in barley cells led to markedly increased haustorial formation rate (i.e., haustorium index) to ∼68%, as compared to ∼52% in the empty vector control (EV) (Figure 3A). By contrast, silencing *CSEP0027* through HIGS significantly decreased haustorium formation rate by ∼40%, relative to the EV control (Figure 3B). Similarly, the silencing of *CSEP0105*, an effector gene used as a positive control (Nowara et al., 2010; Ahmed et al., 2015), led to a stronger effect on the reduction of haustorium index by ∼60%, also relative to the EV (Figure 3B). These data indicate that CSEP0027 contributes to *Bgh* virulence.

The expression of many predicted or functionally confirmed *CSEP* genes is induced during barley infection (Godfrey et al., 2009; Spanu et al., 2010; Pedersen et al., 2012; Hacquard et al., 2013; Schmidt et al., 2014). To further analyze the expression pattern of *CSEP0027*, we conducted a time course experiment (Figure 3C). The transcript level of *CSEP0027* remained low from 0 to12 hpi and was markedly induced at 24 and 48 hpi in both the haustorial containing samples (H) and epiphytic structures (E), with highly enriched transcripts in H sample but not in E sample at 48 hpi (Figure 3C). This expression pattern supports CSEP0027 functioning during barley infection and likely at the post-penetration stages.

### CSEP0027 Interacts With Barley Catalase *Hv*CAT1

**FIGURE 4:**
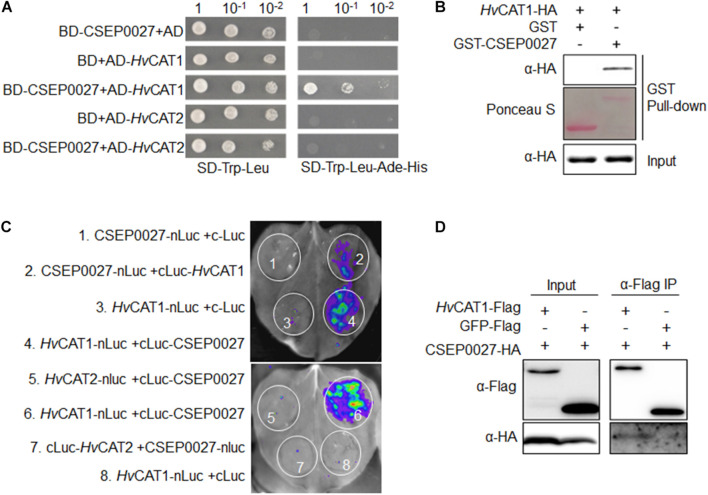
CSEP0027 specifically interacts with barley catalase *Hv*CAT1. **(A)** Yeast two-hybrid (Y2H) assay shows CSEP0027-*Hv*CAT1 interaction. Yeast was transformed with indicated bait and prey constructs. Serial dilutions from cell suspension of yeast expressing bait and prey constructs are shown. Growth on SD-Trp-Leu plates indicates yeast clones carrying the bait and prey constructs. The interactions were detected as yeast growth on SD-Trp-Leu-Ade-His plates. **(B)** Glutathione S-transferase (GST) pull-down assay confirms CSEP0027-*Hv*CAT1 interaction. *Hv*CAT1-HA was extracted from *N. benthamiana* leaves at 2 dpi, while GST-CSEP0027 and GST alone were purified from *E. coli*. GST pull-down fractions were detected by immunoblotting using anti-HA antibody and by Ponceau staining. **(C)** LCI assay confirms CSEP0027-*Hv*CAT1 interaction. The N- or C- terminal fragment of LUC (nLuc or cLuc) was fused with indicated proteins. Indicated fusion pairs were coexpressed in *N. benthamiana* by agroinfiltration. The luminescent signal was collected at 48 hpi with a charge-coupled device (CCD) imaging apparatus. **(D)** Co-immunoprecipiation (Co-IP) analysis validates CSEP0027 and *Hv*CAT1 interaction. *Hv*CAT1-Flag or GFP-Flag was transiently coexpressed with CSEP0027-HA in *N. benthamiana*. The crude proteins were extracted at 48 hpi and subjected to Co-IP analysis.

To identify host targets of CSEP0027, we performed a Y2H screening of a cDNA prey library derived from *Bgh* infected barley leaves. Using a bait of CSEP0027 without the SP, we identified two independent clones harboring the fragments of a barley catalase gene, *HvCAT1*. The targeted Y2H analysis showed that CSEP0027 interacted with full-length *Hv*CAT1 but not with *Hv*CAT2 (Figure 4A), another reported barley catalase that shares more than 70% amino acid identity with *Hv*CAT1 (Supplementary Figure 4; Skadsen et al., 1995). Further interaction analysis indicated that *Hv*CAT1 interacts with CSEP0027 likely through the N-terminal catalase domain but not the C-terminal domain (Supplementary Figure 5).

**FIGURE 5:**
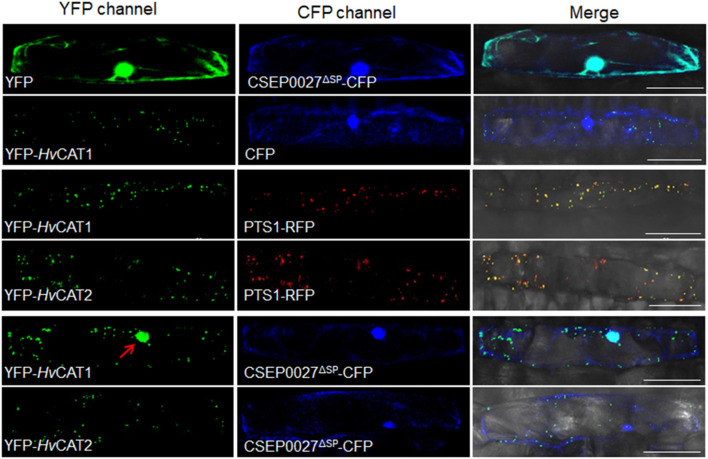
CSEP0027 affects the subcellular localization of *Hv*CAT1. One-week-old barley leaves (P01) were bombarded with combination of indicated constructs coexpressing YFP-*Hv*CAT1/PTS1-RFP, YFP-*Hv*CAT2/PTS1-RFP, YFP-*Hv*CAT1/CSEP0027-CFP, or YFP-*Hv*CAT2/CSEP0027-CFP, respectively. Photographs were taken at 2 days after bombardment using a Nikon confocal laser scanning microscope. Bar = 50 μm.

The interaction between CSEP0027 and *Hv*CAT1 was further verified by *in vitro* and *in vivo* assays (Figures 4B–D). For glutathione S-transferase (GST) pull-down assay, GST-CSEP0027 fusion or GST alone derived from *E. coli* was incubated with *Hv*CAT1-HA containing crude lysate of *N. benthamiana*. An immunoblotting analysis indicated that GST-CSEP0027 pulled down *Hv*CAT1-HA whereas GST did not (Figure 4B). In luciferase complementation imaging (LCI) assays, CSEP0027-nLuc interacted with cLuc-*Hv*CAT1, thus generated luminescence signal, the reciprocal pair *Hv*CAT1-nLuc and cLuc-CSEP0027 also generated strong luminescence signal in *N. benthamiana* (Figures 2, 4), while two pairs of negative control did not produce any detectable signal (Figures 1, 3, 4C). In addition, the *Hv*CAT2-nLuc and cLuc-CSEP0027 did not generate detectable signal (Figures 4C, 5). In co-immunoprecipitation (co-IP) analysis, the *Hv*CAT1-Flag fusion did immuno-precipitate with CSEP0027-HA in *N. benthamiana*, whereas GFP-Flag did not (Figure 4D).

Together, these results indicate that CSEP0027 specifically interacts with barley *Hv*CAT1.

### CSEP0027 Induces the Nuclear Localization of *Hv*CAT1

Since CSEP0027 interacts with *Hv*CAT1, we examined the subcellular localization of CSEP0027 and catalases in barley cells. The plasmids expressing CSEP0027^Δ*SP*^-CFP (Cyan Fluorescent Protein), YFP (Yellow Fluorescent Protein)-*Hv*CAT1, and YFP-*Hv*CAT2 fusions were constructed and delivered into barley cells by particle bombardment. Confocal imaging indicated that CSEP0027^Δ*SP*^-CFP was localized in both cytosol and nucleus, similar to YFP alone (Figure 5, the top panels), while YFP-*Hv*CAT1 was localized in many small dots in the cytoplasm, totally different from that of CFP alone (Figure 5, 2nd panels). Since many plant catalases are localized to peroxisomes, we tested the localization of YFP-*Hv*CAT1 in peroxisomes by coexpression of YFP-*Hv*CAT1 with a peroxisomal marker, PST1-RFP (Red Fluorescent Protein). As expected, YFP-*Hv*CAT1 was almost fully co-localized with PST1-RFP in many cytoplasmic foci in the same cells (Figure 5, 3rd panels). Interestingly, YFP-*Hv*CAT2 was also co-localized with PST1-RFP in most of the cytoplasmic dots (Figure 5, 4th panels). Next, we tested the localization of CSEP0027^Δ*SP*^-CFP and YFP-*Hv*CAT1 in barley cells by coexpression analysis. Remarkably, confocal imaging indicated that YFP-*Hv*CAT1 was detected not only in the peroxisomal dots but also in the nucleus, and CSEP0027^Δ*SP*^-CFP appeared to co-localize with YFP-*Hv*CAT1 in some of the cytoplasmic dots but fully overlapped with YFP-*Hv*CAT1 in the nucleus (Figure 5, 5th panels). Interestingly, when YFP-*Hv*CAT2 was coexpressed with CSEP0027^Δ*SP*^-CFP in barley cells, YFP-*Hv*CAT2 remained to localize in the peroxisomal dots and some dots appeared to overlap with CSEP0027^Δ*SP*^-CFP in the cytoplasm (Figure 5, the bottom panels). These localization analyses suggest that *Hv*CAT1 and CSEP0027 have overlapped subcellular localization in the cytosol and CSEP0027 specifically induces the nuclear localization of *Hv*CAT1.

### *Hv*CAT1 Is Involved in Barley Immunity

**FIGURE 6:**
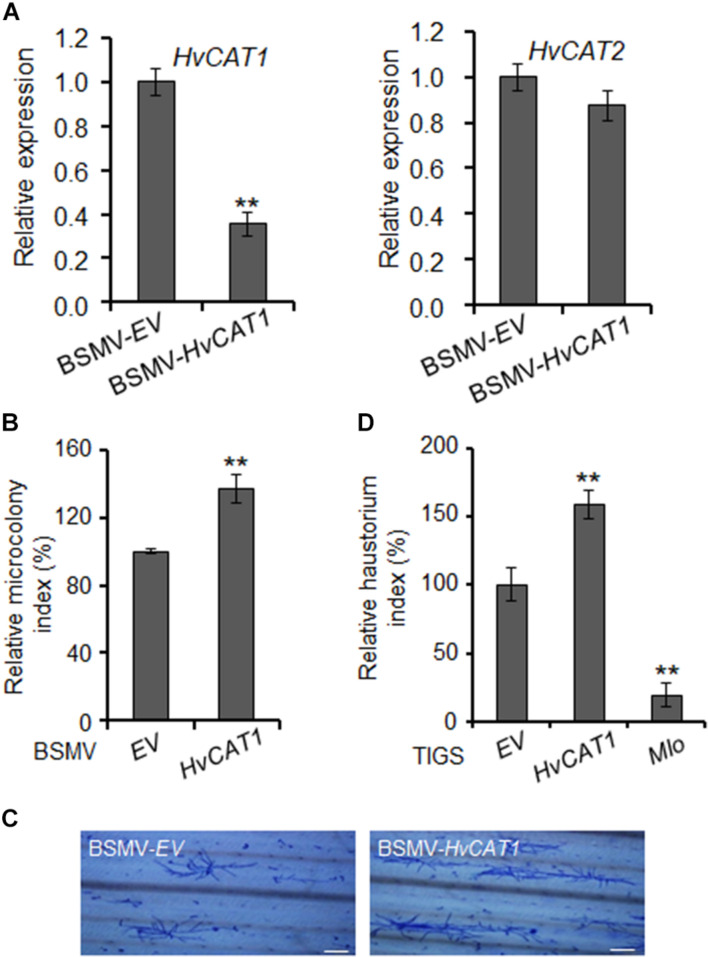
*Hv*CAT1 is involved in barley immunity. **(A)** Barley stripe mosaic virus (BSMV)-*Hv*CAT1 specifically silenced the expression of *HvCAT1* but not *HvCAT2*. *HvCAT1* (left) and *HvCAT2* (right) gene expression levels were determined by qRT-PCR. **(B)** BSMV-VIGS of *HvCAT1* affected barley immunity to *Bgh*. *Bgh* microcolony index was scored upon silencing of *HvCAT1* in barley leaves by using BSMV-*Hv*CAT1 after inoculation with a compatible isolate *BghA6*, and standardized to the BSMV-EV control that was reset to 100%. At least 1,000 interacting sites were microscopically evaluated in one experiment. **(C)** Representative pictures of *Bgh* microcolony and hyphae growth on barley leaf surface in BMSV-VIGS assays. Bar = 200 μm. **(D)** Transiently-induced gene silencing (TIGS) silencing of *HvCAT1* affected barley immunity to *Bgh*. Indicated RNAi construct was bombarded into barley epidermal cells, and *Bgh* haustorium index was scored and standardized to that of EV control. RNAi-*Mlo* construct was used as a control for silencing of *Mlo* that caused dramatic reduction of HI%. The data were presented as average ± SD from three representative replicates. *p < 0.05; **p < 0.01, show significant difference by Student’s *t* test.

The plant catalases play an important role in biotic stress responses by regulating ROS signaling and homeostasis (Du et al., 2008; Chaouch et al., 2010; Sharma and Ahmad, 2014). To evaluate the function of *Hv*CAT1 in barley immunity, we knocked down the *HvCAT1* expression through barley stripe mosaic virus vector (BSMV)-mediated virus-induced gene silencing (VIGS) approach followed by the inoculation of a compatible *Bgh* isolate. An antisense fragment of *HvCAT1* used efficiently silenced *HvCAT1* but not *HvCAT2* (Figure 6A and Supplementary Figure 3). Scoring of *Bgh* microcolony formation rate (i.e., microcolony index, MI%) in barley leaf cells at 60–72 hpi indicated that the relative MI% increased by ∼30% in the *HvCAT1*-silenced leaves as compared to the EV control (Figure 6B). Staining of the *Bgh* infected barley leaves showed more microcolonies and better hyphae growth on the leaf surface of *HvCAT1*-silenced barley, as compared with the EV control (Figure 6C). Furthermore, transiently-induced gene silencing (TIGS) technique was used to silence *HvCAT1* in barley leaf epidermal cells (Himmelbach et al., 2007; Bai et al., 2012). The RNAi-*HvCAT1* construct was delivered into the barley cells by particle bombardment followed by *Bgh* spores inoculation. Relative haustorium formation rate (i.e., relative haustorium index, HI%) scored at 48 hpi also significantly increased by ∼50% as compared with the EV control (Figure 6D). By contrast, TIGS-silencing of the barley *Mlo*, a gene required for full susceptibility to *Bgh* (Kusch and Panstruga, 2017), drastically reduced *Bgh* HI% in barley cells by ∼80% (Figure 6D). Together, these data indicate that *Hv*CAT1 is involved in barley immunity against *Bgh*.

## Discussion

The genomes of many filamentous plant pathogens interacting biotrophically with plants encode hundreds of predicted effectors, and yet loss of function of some individual effectors can have measurable effect on fungal virulence. *B. graminis* fungi also encode several hundreds of CSEPs, and it is expected that many of the CSEPs contribute to the obligate biotrophy life style of the *B. graminis* fungi, for example, co-survival with the host cells or tissues. It is thus of particular interests to understand the functions and mechanisms of CSEPs in regulating host immune responses and cell-death related processes. Here, we identify *Bgh* CSEP0027 that triggers cell death when heterologously expressed in *N. benthamiana*. Importantly, CSEP0027 promotes fungal virulence in barley and interacts with *Hv*CAT1 that is involved in host immunity, most likely, in the maintenance of ROS homeostasis in host cells. In this study, the primary aim in ectopically expressing the *Bgh* CSEPs in *N. benthamiana* is to identify those who may have cell-death related functions, either suppressing or inducing cell death, hoping to better understand the biotrophic lifestyle of the *Bgh* fungus. Indeed, we have predominately identified CSEPs suppressing cell death in *N. benthamiana* (Li et al., 2021), but unexpectedly, CSEP0027 triggering cell death as shown in the present study. We speculate that this cell death activity of CSEP0027 and related pathway may not be fully conserved in dicots and monocots. For example, the co-receptors BAK1 and SOBIR1 are important immune signaling components required for PTI and cell death in dicots (Liu et al., 2016; van der Burgh et al., 2019), while whether the co-receptors are also required for CSEP0027-induced cell death in *N. benthamiana* is not yet resolved here, and importantly, whether the signaling pathway for CSEP0027 induced cell death is shared between *N. benthamiana* and barley awaits for further investigation. Nevertheless, this study findings suggest that *B. graminis* fungi may utilize the CSEPs to target host catalase, a likely component of host ROS networks, presumably to manipulate the ROS homeostasis and signaling for the benefit of the pathogens.

### CSEP0027 Functioning in Fungal Virulence

The well-established HIGS technique has been used for identifying *Bgh* CSEPs with functions in promoting fungal virulence (Nowara et al., 2010). So far, a few dozens of *Bgh* CSEPs have been shown to contribute to *Bgh* pathogenicity (Nowara et al., 2010; Zhang et al., 2012, 2019; Pliego et al., 2013; Aguilar et al., 2015; Ahmed et al., 2015, 2016; Pennington et al., 2019; Li et al., 2021). In the present study, HIGS of *CSEP0027* led to the reduction of HI% by ∼37% in the infected barley cells. Together with the transient overexpression results, our data support the role of CSEP0027 in promoting fungal virulence during barley infection. Our data also suggest that CSEP0027 is most likely a cytoplasmic effector and *Hv*CAT1 is one of its virulence targets. By affecting the subcellular localization of *Hv*CAT1, CSEP0027 may facilitate *Bgh* infection of host barley.

*Bgh CSEP* genes are usually induced and/or differentially expressed during the infection of barley. Some *CSEP* genes are induced at early stages of barley infection, for example, from ∼6 to 12 hpi, whereas others are induced at later stages from 24 to 48 hpi (Godfrey et al., 2009; Zhang et al., 2012; Hackenberg et al., 2013; Schmidt et al., 2014; Aguilar et al., 2015; Ahmed et al., 2015, 2016). *CSEP0027* is induced from 24 to 48 hpi and is enriched in fungal haustoria (Figure 3B). We thus believe CSEP0027 functions at later stages of infection, most likely during and after haustorial formation.

### Regulation of ROS Signaling and Homeostasis

Reactive oxygen species, as major regulatory and signaling molecules, can be generated in different subcellular compartments of plant cells and are regulated by an array of antioxidant systems (Waszczak et al., 2018). During plant–fungus interaction, one of the early events in plant response to fungal penetration is an oxidative burst in the apoplastic space, generated mainly by the phagocyte respiratory burst oxidase homologous nicotinamide adenine dinucleotide phosphate (NADPH) oxidases, cell wall peroxidases, and oxalate oxidases (Hückelhoven, 2007; Lehmann et al., 2015). In barley/wheat and *B. graminis* interactions, H_2_O_2_ and some other ROS molecules are generated in plant cells during the early stages of fungal penetration, participating in the cell wall lignification and apposition (Zhang et al., 1995; Thordal-Christensen et al., 1997; Hückelhoven et al., 1999; Hückelhoven et al., 2001, 2003; Hückelhoven, 2007; Schweizer, 2008; Li et al., 2015). Interestingly, *Bgh* fungus also secrets an extracellular catalase that may function in H_2_O_2_ scavenging in the apoplastic space of host cells (Zhang et al., 2004). The catalases have been known as a class of ROS scavenging enzymes catalyzing the conversion of H_2_O_2_ into H_2_O and O_2_, thereby regulating the homeostasis of the intracellular ROS level (Mhamdi et al., 2010; Sharma and Ahmad, 2014). ROS homeostasis is maintained in a very complex manner, involving different ROS-scavenging enzymes, such as catalases, ascorbate peroxidases, glutathione, superoxide dismutases (Mittler et al., 2004; Torres et al., 2006). The peroxisomal ROS levels are closely regulated by CAT activity, and in Arabidopsis, the primary peroxisomal H_2_O_2_ scavenger is CAT2 (Mhamdi et al., 2012). Here, we show that barley *Hv*CAT1 and *Hv*CAT2 are also peroxisomal catalases. It is unclear whether and when these two *Hv*CAT1 and *Hv*CAT2 are involved in the H_2_O_2_ decomposition and signaling in peroxisomes during barley interaction with *Bgh* fungus. Since CSEP0027 expression is induced and most likely functions post haustorium formation, we speculate that *Hv*CAT1 may play a role in regulating ROS homeostasis at later stages, e.g., during and post haustorium formation. Our preliminary data suggest that CSEP0027 triggered-cell death involves H_2_O_2_ accumulation in *N. benthamiana*, however, it is not clear if the expression CSEP0027 induced disturbance of ROS homeostasis thus cell death, or vice versa. On the other hand, it is also not yet clear if CSEP0027 has activity in cell death during barley interaction with *Bgh* fungus. Undoubtedly, more data are needed for fully understanding the role of CSEP0027 in interacting with barley catalases and in regulating ROS homeostasis during barley interaction with *Bgh* fungus, particularly, the cell death signaling pathway that might be a primary target of the biotrophic fungal pathogen.

The current data are in line with the notion that ROS, in particular cellular H_2_O_2_, may play an important role in the barley interactions with the *B. graminis* fungi. It is not unexpected that peroxisomal ROS signaling/homeostasis and ROS signaling cross-talk among the organelles are integral and important parts of barley defense responses to the biotrophic *Bgh* fungal pathogen.

### The Regulation of Plant Catalases

Apart from being regulated at transcriptional level, plant catalases are also regulated at post-translational level (Mhamdi et al., 2010). A variety of plant proteins have been reported to affect the activity and stability of plant catalases (Yang and Poovaiah, 2002; Fukamatsu et al., 2003; Verslues et al., 2007; Li et al., 2013, 2015; Zou et al., 2015; Kneeshaw et al., 2017). In addition, some pathogen secreted proteins are also identified to interact with the plant catalases and affect their activity, stability, and subcellular localization (Inaba et al., 2011; Mathioudakis et al., 2013; Zhang et al., 2015; Murota et al., 2017; Sun et al., 2017). In line with these examples, the current study data provide new evidence that biotrophic fungal pathogen also secretes an effector to target and affect host catalase subcellular localization in plants.

The plant catalases are mostly peroxisomal proteins and imported into the peroxisome matrix *via* the peroxisomal targeting signal 1 (PTS1) pathway, i.e., relying on the C-terminal tripeptide PTS1 signal to interact with a peroxisomal receptor and translocate into the peroxisome (Gatto et al., 2000; Lanyon-Hogg et al., 2010). Barley *Hv*CAT1 and *Hv*CAT2, each contains a typical PTS1 signal, PNM or PSM, respectively (Supplementary Figure 4; Mhamdi et al., 2012), and both are localized to the peroxisomes of barley cells in a transient expression analysis (Figure 5). Although different mechanisms may account for the specific re-localization of *Hv*CAT1 upon co-expression with CSEP0027, one scenario can be that CSEP0027 interacts with *Hv*CAT1 but not *Hv*CAT2 in the cytoplasm thus interferes with the interaction of PTS1 signal of *Hv*CAT1 with the peroxisomal receptor. However, how *Hv*CAT1 is specifically regulated by CSEP0027 is not yet clear. Further investigation of the subcellular localization, trafficking, and post-translational modification of *Hv*CAT1 will help to better understand the functions of the catalase and the virulence strategies of the biotrophic fungus.

## Materials and Methods

### Plant and Fungal Materials

Barley (*Hordeum vulgare* L.) cultivars (cv) in this study include Golden Promise and “P01” (isogenic line from cv Pallas containing *Mla1*). Barley seedlings were grown in a growth chamber at 20°C with 16 h light and 8 h dark cycles. *N. benthamiana* plants were grown in greenhouse at 24 ± 1°C with a long-day cycle (16 h light/8 h dark).

The barley powdery mildew (*B. graminis* f.sp. *hordei* [*Bgh*]) isolates A6 (*AvrMla6, AvrMla10*, and *virMla1*) and K1 (*AvrMla1*, *virMla6*, and *virMla10*) used in this study were maintained on Golden Promise.

### Cloning and Plasmid Construction for *CSEP* Genes

Total RNA was extracted from P01 barley leaves inoculated with *Bgh* isolate A6 using Trizol solution (Invitrogen; 15596-026) and the cDNA was synthesized using reverse transcriptase M-MLV (Invitrogen; C28025). Candidate *CSEP* sequences excluding the signal peptide (ΔSP) were amplified using the specific primer pairs (Supplementary Table 2) and subcloned into pGR107 vector through restriction enzyme digestion and ligation for agroinfiltration in *N. benthamiana* (Wang et al., 2011), all candidates confirmed by sequencing.

### Quantitative Real-Time Polymerase Chain Reaction (qRT-PCR)

The analysis of *CSEP0027* expression profile was performed as previously described (Ahmed et al., 2015). In brief, total RNA was isolated from P01 leaves at 0, 3, 6, 12, 24, and 48 hpi inoculated with virulent isolate A6. The epiphytic *Bgh* tissues and the remaining leaf tissues containing *Bgh* haustoria were separately collected at 24 and 48 hpi. The epiphytic tissues were collected from leaf surfaces by dipping the *Bgh*-infected leaves into 10% cellulose acetate according to previously described (Ahmed et al., 2015). A quantitative real-time PCR (qRT-PCR) was performed on Applied Biosystems step-one real time PCR system with indicated primers (Supplementary Table 2). *Bgh* glyceraldehyde 3-phosphate dehydrogenase was used as the reference gene. The statistical significance was evaluated by Student’s *t* test. The assays were repeated two times with three replicates each time.

### Yeast Invertase Secretion Assay

The yeast invertase secretion was previously described (Gu et al., 2011). Briefly, the predicted SP sequence of CSEP0027 and Avr1b, or the first 25 amino acids of *Magnaporthe oryzae* Mg87 were fused in frame with the yeast invertase lacking its own SP in the vector pSUC2. The pSUC2-derived constructs were transformed into the invertase secretion-deficient yeast strain YTK12, and yeasts were then placed on CMD-W medium (0.67% yeast N base without amino acids, 0.075% tryptophan dropout supplement, 2% sucrose, 0.1% glucose, and 2% agar). The positive yeast clones were transferred onto YPRAA medium (1% yeast extract, 2% peptone, 2% raffinose, 2 μg L^–1^ antimycin, and 2% agar) for growth testing. Invertase activity was also detected by monitoring conversion of TTC to the insoluble red-colored triphenylformazan.

### Single-Cell Transient Gene Expression and Silencing Assay

Single-cell transient gene expression assay was carried out as previously described (Bai et al., 2012). Briefly, a construct expressing a gene-of-interest was bombarded in barley leaf epidermal cells together with a vector expressing ß-glucuronidase (GUS) reporter. The leaves were inoculated with a compatible *Bgh* isolate at 4 h after the bombardment and then stained with GUS staining solution at 48 hpi. The fungal haustorium index was scored as previously described in the barley leaves after inoculated with *Bgh* spores. The statistical significance was evaluated by Student’s *t* test with data from three replicate experiments that have been repeated for three times.

For transient gene silencing assay, the specific gene fragments were cloned into pIPK007 to form a hairpin structure and expression driven by 35S promoter as previously described (Himmelbach et al., 2007). The remaining steps were the same as the transient gene expression assay, except that leaves were inoculated with *Bgh* isolates at 48 h after bombardment.

### Y2H Analysis

Yeast two-hybrid screening was performed according to the protocols of the manufacturer (Clontech; PT4048-1). In total, 5 × 10^7^ transformants were screened. In brief, yeast strain Y2HGold expressing *pGBKT7-CSEP0027* (ΔSP) was used for mating with yeast strain Y187 harboring a cDNA prey library derived from *Bgh*-infected barley leaves and placed onto SD-Leu-Trp-His-Ade plates at 30°C. After 35 days, the resistant clones were selected for further verification.

For Y2H assay, the corresponding bait and prey vectors were co-transformed into yeast strain Y2HGold and plated onto SD-Leu-Trp plates. The positive interactions were detected by placing the strains onto SD-Leu-Trp-His-Ade plates at 30°C.

### Luciferase Complementation Imaging Assays

Luciferase complementation imaging assays were performed according to previously described by Chen et al. (2008). Briefly, the coding region of *CSEP0027* (ΔSP) and *Hv*CAT1 were subcloned into vectors pCAMBIA-Cluc or pCAMBIA-Nluc, respectively, to generate constructs for expressing Cluc-CSEP0027 and Cluc-*Hv*CAT1, or CSEP0027-Nluc and *Hv*CAT1-Nluc. The NLuc-/CLuc-derivative constructs were transformed into the *A. tumefaciens* strain GV3101. The overnight agrobacteria cultures were resuspended with infiltration buffer (2% sucrose, 0.5% MS, 100 μM acetosyringone, and 10 mM MES) into OD600 = 1.0. Equal volume of agrobacteria resuspension carrying the nLUC and cLUC derivative constructs were mixed and co-infiltrated into the *N. benthamiana* leaves. The infiltrated area was examined for the luciferase activity 40–50 h post agroinfiltration with a cooled charge-coupled device (CCD) imaging apparatus. For each pair of constructs, at least 10 leaves were co-infiltrated in one experiment, and three independent replicates were conducted.

### Glutathione S-Transferase(GST) Pull-Down and Co-immunoprecipitation (Co-IP) Assays

Pull-down assays were performed according to previously described with some modifications (Chang et al., 2013). Briefly, 500 ng of GST-CSEP0027 and GST proteins purified from *Escherichia coli* were incubated with 150 μl of Glutathione Sepharose 4B beads for 1 h at 4°C, then, beads were sealed with 100 μg BSA for 1 h and incubated with 1.0 g crude protein extracted from *N. benthamiana* leaves expressing *Hv*CAT1-HA. After incubation for 2 h, the beads were washed five times with RB buffer, then resuspended with 30 μl of 2 × Laemmli buffer, and loaded for sodium dodecyl-sulfate polyacrylamide gel electrophoresis (SDS-PAGE) and immunoblotting with anti-HA antibody. GST-CSEP0027 and GST proteins were detected by Ponceau staining.

For Co-IP assay, the total proteins extracted from *N. benthamiana* coexpressing GFP-Flag/CSEP0027-HA or *Hv*CAT1-Flag/CSEP0027-HA were incubated with anti-FLAG antibody-coupled beads for 2 h, then washed five times with extraction buffer, proteins were further eluted from the beads using 0.5 mg ml^–1^ 3 × Flag peptide and used for immunoblotting with anti-HA antibody, or anti-Flag antibody.

### Confocal Laser Scanning Microscopy and Localization Analysis

For subcellular localization analysis, the coding sequences of *CSEP0027*^Δ*SP*^, *HvCAT1* and *HvCAT2* were subcloned into vectors pUBI-mYFP-GW and pUBI-GW-CFP to generate pUBI-CSEP0027 ^Δ*SP*^-CFP, pUBI-mYFP-*Hv*CAT1, and pUBI-mYFP-*Hv*CAT2 constructs. A pair of constructs was delivered into barley leaf epidermal cells by the particle bombardment for coexpression of the indicated fusion proteins, and confocal imaging was conducted at 48 h post-particle delivery. Laser illumination was set at 405 nm for CFP, 488 nm for YFP, and 561 nm for RFP using a Nikon confocal microscope. This assay was repeated three independent times and at least 20 cells were examined for each coexpression.

### Barley Stripe Mosaic Virus (BSMV)-Mediated Gene Silencing in Barley

Barley stripe mosaic virus-mediated gene silencing in barley was performed as previously described (Yuan et al., 2011). Briefly, an antisense fragment of *HvCAT1* was cloned into the pCaBS-γbLIC vector to create pCaBS-γb-*Hv*CAT1 construct with indicated primers (Supplementary Table 2). pCaBS-α, pCaBS-β, and pCaBS-γb-*Hv*CAT1 constructs were transformed into *A. tumefaciens* strain EHA105, respectively. The agrobacteria were resuspended in infiltration buffer to OD_600_ = 1.0 and mixed at 1:1:1 ratio to infiltrate *N. benthamiana*. After 12 days, *N. benthamiana* leaf sap was extracted to inoculate 10-day-old barley leaves. After 2–3 weeks of inoculation, the newly emerged leaves with virus caused symptoms were used for *Bgh* infection, and microcolony scoring was done at ∼60–72 hpi. For each treatment, at least four barley leaves were chosen for analysis, and three independent replicates were conducted. The statistical significance was evaluated by Student’s *t* test.

### Agroinfiltration Mediated Transient Gene Expression in *N. benthamiana*

*Agrobacterium tumefaciens*-mediated transient gene expression in *N. benthamiana* assays were performed as described previously (Wang et al., 2011). *A. tumefaciens* strain GV3101 was transformed with indicated constructs. Agrobacteria were cultured overnight at 28°C, at 200 rpm, then resuspended in 10 mM MgCl_2_ to a final OD_600_ = 0.5 and infiltrated into 4-week-old *N. benthamiana* leaves. The cell death symptoms were photographed at 5 days post-infiltration. For trypan blue staining, the leaves were boiled in a 1:1 mixture of ethanol and staining solution for 5 min as described before (Bai et al., 2012). The leaves were de-stained with chloral hydrate solution (2.5 g ml^–1^) for 2 days.

## Data Availability Statement

The original contributions presented in the study are included in the article/Supplementary Material, further inquiries can be directed to the corresponding author.

## Author Contributions

HY and Q-HS designed the research. HY, CJ, and HP performed the experiments with helps from XL, JL, and WH. HY, CJ, LZ, XL, RF, and Q-HS, analyzed the data. WL and RF provided the reagents. Q-HS, HY, and LZ wrote the manuscript. All authors contributed to the article and approved the submitted version.

## Conflict of Interest

The authors declare that the research was conducted in the absence of any commercial or financial relationships that could be construed as a potential conflict of interest.

## Publisher’s Note

All claims expressed in this article are solely those of the authors and do not necessarily represent those of their affiliated organizations, or those of the publisher, the editors and the reviewers. Any product that may be evaluated in this article, or claim that may be made by its manufacturer, is not guaranteed or endorsed by the publisher.

## Supplementary Material

Supplementary Figure 1 The MLA13/AVR_*a13*_ triggers cell death in *N. benthamiana.* Expression of *MLA13/AVR_*a13*_* and *GFP* in *N. benthamiana*. The experimental procedure used here was the same as that in Figure 1A.

Supplementary Figure 2 CSEP0027 induces H_2_O_2_ production in *N. benthamiana.* DAB staining was performed at 2 days after infiltration to detect H_2_O_2_ accumulation in the areas infiltrated with agrobacteria transformed with a corresponding construct.

Supplementary Figure 3 *Bgt* homologs of CSEP0027 do not trigger cell death in *N. benthamiana.* **(A)** Protein sequence alignment of CSEP0027 and its *Bgt* homologs. Alignment was performed using the DNAMAN software. The red box indicates signal peptides, blue box indicates the Y/FxC motif, and green box indicates a C-terminal conserved cysteine. **(B)** Expression of *CSEP0027* and its *Bgt* homologs in *N. benthamiana*. The experimental procedure used here was the same as that in Figure 1A.

Supplementary Figure 4 Sequence alignment of amino acids of *Hv*CAT1 and *Hv*CAT2.

Supplementary Figure 5 CSEP0027 N-terminus interacts with *Hv*CAT1 in yeast. **(A)** Schematic diagram of *Hv*CAT1 constructs, and *Hv*CAT1-NT (1–401) and *Hv*CAT1-CT (402–492) used in the Y2H assay. Catalase core domain and catalase-related immune responsive domain (catalase-rel) are indicated. **(B)** Y2H analysis of the interaction between NT- or CT-fragments of *Hv*CAT1 and CSEP0027. Yeast was transformed with the indicated bait and prey constructs. Serial dilutions from cell suspension of yeast expressing bait and prey constructs are shown. Growth on SD-Leu-Trp plates indicates yeast clone carrying bait and prey constructs. Interactions were detected on SD-Leu-Trp-His-Ade plates.
