# Supplementary figures and images for: The Powdery Mildew Effector CSEP0027 Interacts With Barley Catalase to Regulate Host Immunity
Source: Front Plant Sci. 2021 Sep 9;12:733237. doi: 10.3389/fpls.2021.733237 (PMC8458882; doi:10.3389/fpls.2021.733237)

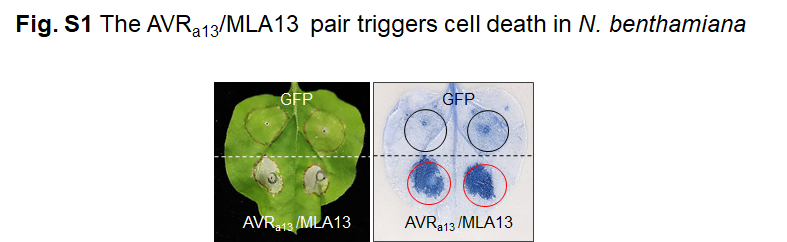

Supplement: Supplementary Figure 1 — The MLA13/AVRa13 triggers cell death in N. benthamiana. Expression of MLA13/AVRa13 and GFP in N. benthamiana. The experimental procedure used here was the same as that in Figure 1A. [file Image_1.TIF]

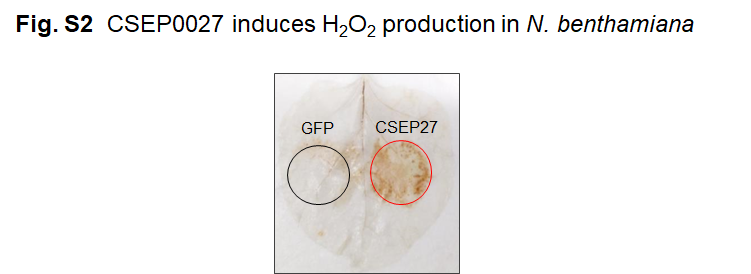

Supplement: Supplementary Figure 2 — CSEP0027 induces H2O2 production in N. benthamiana. DAB staining was performed at 2 days after infiltration to detect H2O2 accumulation in the areas infiltrated with agrobacteria transformed with a corresponding construct. [file Image_2.TIF]

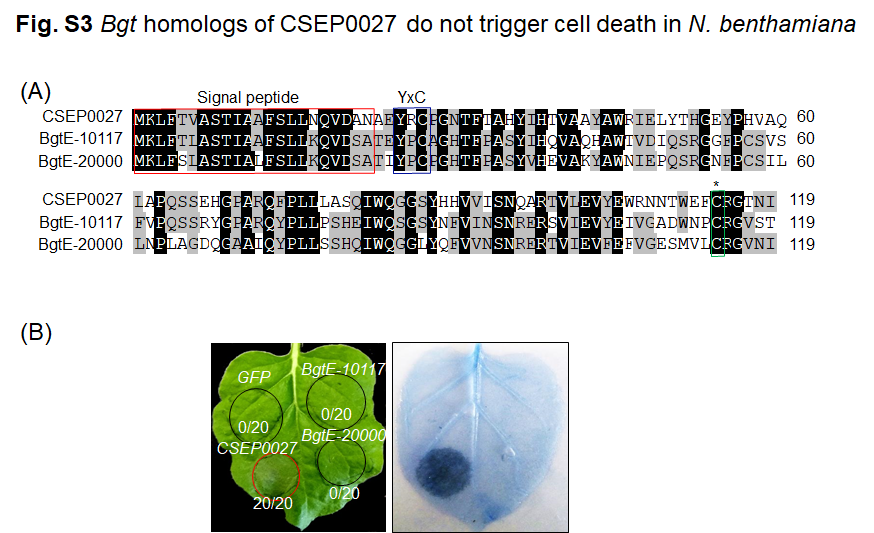

Supplement: Supplementary Figure 3 — Bgt homologs of CSEP0027 do not trigger cell death in N. benthamiana. (A) Protein sequence alignment of CSEP0027 and its Bgt homologs. Alignment was performed using the DNAMAN software. The red box indicates signal peptides, blue box indicates the Y/FxC motif, and green box indicates a C-terminal conserved cysteine. (B) Expression of CSEP0027 and its Bgt homologs in N. benthamiana. The experimental procedure used here was the same as that in Figure 1A. [file Image_3.TIF]

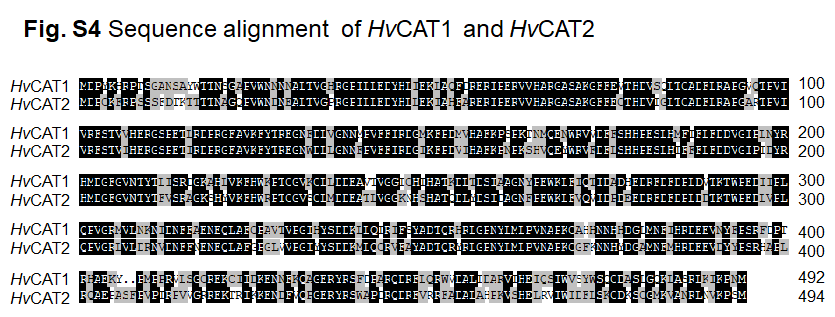

Supplement: Supplementary Figure 4 — Sequence alignment of amino acids of HvCAT1 and HvCAT2. [file Image_4.TIF]

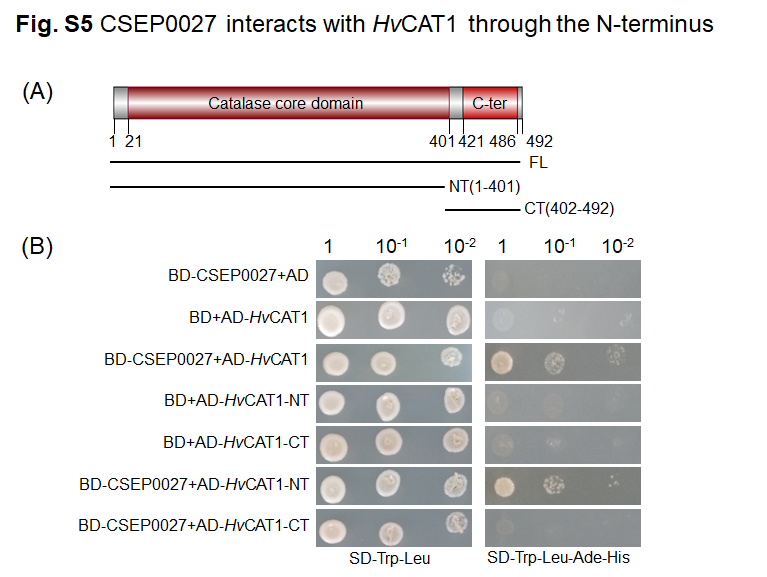

Supplement: Supplementary Figure 5 — CSEP0027 N-terminus interacts with HvCAT1 in yeast. (A) Schematic diagram of HvCAT1 constructs, and HvCAT1-NT (1–401) and HvCAT1-CT (402–492) used in the Y2H assay. Catalase core domain and catalase-related immune responsive domain (catalase-rel) are indicated. (B) Y2H analysis of the interaction between NT- or CT-fragments of HvCAT1 and CSEP0027. Yeast was transformed with the indicated bait and prey constructs. Serial dilutions from cell suspension of yeast expressing bait and prey constructs are shown. Growth on SD-Leu-Trp plates indicates yeast clone carrying bait and prey constructs. Interactions were detected on SD-Leu-Trp-His-Ade plates. [file Image_5.TIF]
