# Supplementary material for: The Powdery Mildew Effector CSEP0027 Interacts With Barley Catalase to Regulate Host Immunity
Source: Front Plant Sci. 2021 Sep 9;12:733237. doi: 10.3389/fpls.2021.733237 (PMC8458882; doi:10.3389/fpls.2021.733237)
Supplement: Supplementary file 6 [file Data_Sheet_1.PDF]

Table S1 List of *Bgh CSEP* genes amplified and screened for cell death phenotypes

Family[a]	name
4	*CSEP0036*, *CSEP0126*, *CSEP0154*
5	*CSEP0099*, *CSEP0112*, *CSEP0442*, *CSEP0145*
6	*CSEP0006*, *CSEP0175*, *CSEP0176*, *CSEP0230*, *CSEP0397*, *CSEP0398*, *CSEP0434*, *CSEP0466*, *CSEP0467*
8	*CSEP0147*, *CSEP0148*, *CSEP0150*
12	*CSEP0081*, *CSEP0090*, *CSEP0091*, *CSEP0092*, *CSEP0094*, *CSEP0197*
13	*CSEP0167*, *CSEP0168*, *CSEP0169*, *CSEP0471*
15	*CSEP0341*, *CSEP0388*, *CSEP0418*, *CSEP0432*
17	*CSEP0387*
19	*CSEP0139*, *CSEP0221*, *CSEP0360*
20	*CSEP0063*, *CSEP0101*, *CSEP0448*
21	*CSEP0066*, *CSEP0486*, *CSEP0264*
22	*CSEP0137*, *CSEP0146*, *CSEP0180*
23	*CSEP0220*, *CSEP0248*, *CSEP0363*, *CSEP0443*
24	*CSEP0001*, *CSEP0104*, *CSEP0111*, *CSEP0133*
25	*CSEP0005*, *CSEP0012*, *CSEP0086*, *CSEP0098*
29	*CSEP0059*, *CSEP0060*, *CSEP0100*
30	*CSEP0071*
31	*CSEP0257*, *CSEP0481*
32	*CSEP0106*, *CSEP0109*
34	*CSEP0371*, *CSEP0372*, *CSEP0373*, *CSEP0374*
35	*CSEP0380*, *CSEP0488*, *CSEP0379*, *CSEP0489*
36	*CSEP0004*
41	*CSEP0027*, *CSEP0028*, *CSEP0340*
43	*CSEP0122*
44	*CSEP0074*, *CSEP0075*
46	*CSEP0083*, *CSEP0084*, *CSEP0085*
47	*CSEP0311*
49	*CSEP0177*
59	*CSEP0057*
61	*CSEP0123*, *CSEP0140*
63	*CSEP0136*
64	*CSEP0141*
66	*CSEP0262*, *CSEP0485*
70	*CSEP0361*
n.d.	*CSEP0043*, *CSEP0048*, *CSEP0182*, *CSEP0224*, *CSEP0273*, *CSEP0381*, *CSEP0474*

[a] family number is assigned according to Pedersen et al., 2012
