# Supplementary material for: The Powdery Mildew Effector CSEP0027 Interacts With Barley Catalase to Regulate Host Immunity
Source: Front Plant Sci. 2021 Sep 9;12:733237. doi: 10.3389/fpls.2021.733237 (PMC8458882; doi:10.3389/fpls.2021.733237)
Supplement: Supplementary file 7 [file Data_Sheet_2.PDF]

Table S2 List of primers used in this study

Primer	Sequence (5'-3')	Construct
Y90-CSEP0027_F	caccatcgatatgaatgcagaatatcgctgtc	pGR107-CSEP0027-HA
Y91-HA-_R	ctacgcatagtcaggaacatcgtatgggtaagaaccaatattcgtgcctctgcaaa	
Y92-CSEP0028_F	caccatcgatatggacggctttgattgcaatg	pGR107-CSEP0028-HA
Y93-CSEP0028_R	cgcgtcgaccgcatagtcaggaacatcgtatgggtaagaacctgcactcacacattcata	
Y94-CSEP0340_F	caccatcgatatgctagagtattttgactgcc	pGR107-CSEP0340-HA
Y95-CSEP0340_R	cgcgtcgaccgcatagtcaggaacatcgtatgggtaagaaccgccatttacaacgtcgcat	
Y207-CSEP0027-SP_F	ggggaattcatgaaacttttcaccgttgcc	pSUC2-CSEP0027-SP
Y208-CSEP0027-SP_R	gggctcgagtgcattagcatctacttggtt	
Y213-attb-CSEP0027_F	ggggacaagtttgtacaaaaaagcaggctcttttactgcccattatatac	RNAi-CSEP0027
Y214-attb-CSEP0027_R	ggggaccactttgtacaagaaagctgggtcttcctccattcgtatacttc	
Y217-CSEP0027_F	atctcagaggaggacctgcatatgatgaatgcagaatatcgctgtc	pGBKT7-CSEP0027
Y218-CSEP0027_R	aggtcgacggatccccgggaattcttaaatattcgtgcctctgcaaa	
Y219-HvCAT1_F	gacgtaccagattacgctcatatgatggatccctacaagcaccg	pGAD-HvCAT1
Y220-HvCAT1_R	tcgatgcccacccgggtggaattcttacatgttcggcttaatcttg	
Y221-HvCAT2_F	gacgtaccagattacgctcatatgatggatccctgcaagttccgg	pGAD-HvCAT2
Y222-HvCAT2_R	tcgatgcccacccgggtggaattcatccatggcagatccatcca	
Y223-HvCAT1$_{1203}$_R	tcgatgcccacccgggtggaattcttaacgagtaggatcaaaccttga	pGAD-HvCAT1_NT
Y224-HvCAT1$_{1204}$_F	gacgtaccagattacgctcatatgatgcatgctgaaaagtaccctatg	pGAD-HvCAT1_CT
Y235-CSEP0027-nluc_F	ggagagaacacgggggacgagctcgatgaatgcagaatatcgctgtc	CSEP0027-nLuc
Y236-CSEP0027-nluc_R	ggacgcgtacgagatctggtcgacaatattcgtgcctctgcaaa	
Y239-CSEP0027-cluc_F	tcccggggcggtacccggggatccatgaatgcagaatatcgctgtc	Cluc-CSEP0027
Y240-CSEP0027-cluc_R	atgatacgaacgaaagctctgcaggttaaatattcgtgcctctgca	
Y241-HvCAT1-nluc_F	ggagagaacacgggggacgagctcatggatccctacaagcaccg	HvCAT1-nLuc

Y242-HvCAT1-nluc_R	ggacgcgtacgagatctggtcgaccatgttcggcttaatcttg	
Y243-HvCAT1-cluc_F<br>Y244-HvCAT1-cluc_R	tcccggggcggtacccggggatccatggatccctacaagcaccg<br>atgatacgaacgaaagctctgcaggttacatgttcggcttaatcttg	Cluc-HvCAT1
Y245-HvCAT2-nluc_F<br>Y246-HvCAT2-nluc_R	ggagagaacacgggggacgagctcatggatccctgcaagttccgg<br>ggacgcgtacgagatctggtcgaccatgcttggcttcacgttga	HvCAT2-nLuc
Y247-HvCAT2-cluc_F<br>Y248-HvCAT2-cluc_R	tcccggggcggtacccggggatccatggatccctgcaagttccg<br>atgatacgaacgaaagctctgcaggtcacatgcttggcttcacgt	Cluc-HvCAT2
HvCAT1_F<br>HvCAT1-Flag_R	gggatcgatatggatccctacaagcaccg<br>ctatttatcatcgtcatccttataatcagaacccatgttcggcttaatcttgag	HvCAT1-Flag
HvCAT2_F<br>HvCAT2-Flag_R	caccatcgat**atg**gatccctgcaagttccgg<br>ctatttatcatcgtcatccttataatcagaacccatgcttggcttcacgttgag	HvCAT2-Flag
NbCAT2-Flag_R	gatcgatatggattataaggatgacgatgataaaggtatgaatccatacaagtatcgtc	
Y261-BSMV-CAT1_F<br>Y262-BSMV-CAT1_R	aaggaagtttaaccaagtccgaagaccaacat<br>aaccaccaccaccgtcgtgatcagcatcaatggtc	BSMV-*Hv*CAT1
CSEP0027-qPCR_F<br>CSEP0027-qPCR_R	agcctatgcctggagaat<br>cagtcctagcctgatttg	qRT-PCR primer
BghGAPDH_F<br>BghGAPDH_R	atgaactacaaggcatcctgtca<br>accatgcgactagcttaacaaag	qRT-PCR primer
Y263-HvCAT1_F<br>Y264-HvCAT1_R	cctccgcgtgttctatctgg<br>tcatgggtgacacgagcatc	qRT-PCR primer
Y265-HvCAT2_F<br>Y266-HvCAT2_R	ctctacgactccatcgacgc<br>agctgctcgttctcgttgaa	qRT-PCR primer
Y267-HvUBI_F<br>Y268-HvUBI_R	accctcgccgactacaacat<br>cagtagtggcggtcgaagtg	qRT-PCR primer
Y659-CSEP0027_F<br>Y272-CSEP0027_R	cggggatccatgaatgcagaatatcgctgtc<br>cgcgtcgacttaaatattcgtgcctctgcaaa	pGEX4T-CSEP0027
Y269-TIGS-CAT1_F<br>Y270-TIGS-CAT1_	ggggacaagtttgtacaaaaaagcaggcttcccaagtccgaagaccaacat<br>ggggaccactttgtacaagaaagctgggtccgtgatcagcatc	RNAi-*Hv*CAT1

R	aatggtc	
attb-HvCAT1_F	ggggacaagtttgtacaaaaaagcaggcttcatggatccctaca agcaccg	pUbi-mYFP-HvCAT1
attb-HvCAT1_R	ggggaccactttgtacaagaaagctgggtccatgttcggcttaat cttgag	
attb-HvCAT2_F	ggggacaagtttgtacaaaaaagcaggcttcatggatccctgca agttccgg	pUbi-mYFP-HvCAT2
attb-HvCAT2_R	ggggaccactttgtacaagaaagctgggtccatgcttggcttca cgttgag	
attb-CSEP0027_F	ggggacaagtttgtacaaaaaagcaggcttcatgcagaatatcg ctgtc	pUbi-CSEP0027-CFP
attb-CSEP0027-N_ R	ggggaccactttgtacaagaaagctgggtcaatattcgtgcctct gcaaa	
attb-CSEP0027_R	ggggaccactttgtacaagaaagctgggtcttaaatattcgtgcc tctgcaaa	pUbi-CSEP0027
BgtE-10117_F	caccatcgatatggctacagaatatccctgtgct cagtcctagcctgatttg	pGR107-BgtE10117
BgtE-10117_R	ggggtcgactcaagtgctcacgcccctgca	
BgtE-20000_F	caccatcgatatggctacaatatatccctgtcc	pGR107-BgtE20000
BgtE-20000_R	ggggtcgactcaaatgttaacgcccctgca	
Y209-pPLW2_F	ctcgaattcgattgggtaccgcgtcagtga	pPWL2:CSEP0027-FL:mC herry:NLS + BAS4:EGFP
Y210-pPLW2_R	cattttgaaagtttttaatt	
211-pPLW2R-CSE P0027_F	aattaaaaactttcaaaatgaaacttttcaccgttgcc	
Y212CSEP0027_R	gacagatctaatattcgtgcctctgcaaaa	
